# Supplementary material for: Treatment outcome of neonatal sepsis and associated factors among neonates admitted to neonatal intensive care unit in public hospitals, Addis Ababa, Ethiopia, 2021. Multi-center cross-sectional study
Source: PLoS One. 2023 May 30;18(5):e0284983. doi: 10.1371/journal.pone.0284983 (PMC10228817; doi:10.1371/journal.pone.0284983)
Supplement: Attachment — Submitted filename: comments to authors.docx [file pone.0284983.s001.docx]

**Comments to authors**

1. **On the title and abstract part:**

The title needs modification and be more specific because it just measured the factors contributing to the poor treatment due to neonatal sepsis, it also should be specific outcome either mortality, complications, worsened and so on among the poor outcomes.

In abstract, the main gap in knowledge is missing; the aims and the rationale of the study have not been explicitly mentioned.

The method section is not clear and adequate. In conclusion, the sentence "Gestational age, grunting, meconium amniotic stained, hypertensive, meropenum and C-reactive protein level were significantly associated with treatment outcome of neonatal sepsis" is not clear, which treatment outcome? Which is the most important finding of this study? The recommendation of the results of the study is missed.

2. **In the introduction part:**

The way the introduction has been written is not good, it is better to include risk factors contributing to poor treatment outcome. It talks about specifically mortality not poor outcome Also, the main gap in the knowledge and the rationales of the study are missed.

3. **Material and Methods:**

The method part is not clear and lacking many things such as; Inclusion criteria are not mentioned, algorithm for sample allocation, sepsis diagnosis and treatment guidelines in NICU among the four specific hospital.

4. **Result part:**

The results of this study lacks many things are missed such as clinical parameters, types of sepsis, neonatal related characteristics, laboratory findings, comorbidity, and medication related factors,

Etiology of sepsis is entirely missed.

The result among four hospitals are not separately described

5. **Discussion**

The discussion section is poor, the possible justification is also poor.

It should be re-written in more coherent and concise way with logical explanation

6. **Conclusion and recommendation**

The conclusion is not written strongly. The recommendation is totally missed.

7. **Figures and tables**

Table-2 should be divided in to two tables; some findings should be described with figures

8. **Does the paper raise any concerns?**

There are no major concerns raised by the paper.

There seems no ethical concern.

Under statistical analysis, why did you do logistic regression if your primary outcome is mortality among poor treatment outcome, rather cox regression is preferred.

References need some modifications that for some reference there is ‘et al’ where as none for some reference.

9. **Competing interest**

There is no competing interest

10. **English editing**

The manuscript requires English editing to correct the grammar or flow.

**Recommendations to the Editor:**

Better to modify the manuscript as per the comments above

No additional comment
